# Scarlet Fever

**Published:** 1892-08-27

**Authors:** J. Brendon Curgenven

**Affiliations:** Teddington Hall


### Editor's Letter-Box.

[Our correspondents are reminded that prolixity is a great bar to publication, and that brevity of style and conciseness of statement greatly facilitate early insertion.]

### SCARLET FEVER.

Sir,—A short time since I received a statement from Mr. Monro, the Sanitary Inspector of the Enfield Board of Health, on the results obtained from supplying the Eucalyptus Disinfectant for inunction in 54 cases of scarlet fever amongst the poor in houses where there were other children. Directions were given for the inunction to be carried out, as advised by me in my paper on "Disinfection of Scarlet Fever," and, although some of the mothers did not do it efficiently because the children objected to the smell, 43 of the primary cases were not followed by a second. In the other eleven, three occurred on the sixth and seventh days after the first, and were most probably infected before the use of the disinfectant; the other eight occurred from two to eight weeks after the first. Most of them may have been infected from other sources, as an epidemic was then prevailing, and all of them occurred after the disinfectant was discontinued. The above shows a percentage of 20 fresh cases occurring in houses within eight weeks, where the Eucalyptus was used, but with little medical supervision. At Nottingham, Dr. Bobbyer stated lately that in 100 primary hospital cases, 23 per cent. additional cases occurred in the houses from which they were removed within three weeks, and for every 100 home cases treated nearly 60 per cent. additional ones occurred. The superiority of this imperfect trial of disinfection by Eucalyptus inunction is thus shown to be 40 per cent. better than any other form of home disinfection, and 3 per cent. better than that obtained by prompt removal of the cases to hospital and the disinfection of their homes. If the Enfield cases were limited to those that occurred within four weeks after the first, as there was one at five weeks and two at eight weeks' interval, then only 14 per cent. of additional cases occurred, or 9 per cent. better than under the present system of removal; and if the three already infected cases are excluded, the fresh cases are reduced to 9 per cent. From all the reports that I receive from medical men, who carefully carry out this method of disinfection on the cases and on the children that have been exposed to the infection, it is seldom that a second case occurs, and from my own experience a second case does not occur except in those already infected.—Yours faithfully, J. Brendon Curgenven.

Teddington Hall, August 15th.

